# Physical activity among adolescents and young adults living with chronic pain and sickle cell disease: a qualitative examination

**DOI:** 10.1093/jpepsy/jsaf055

**Published:** 2025-07-22

**Authors:** Jan T Mooney, Cynthia Sinha, Nitya Bakshi, Anjanette Nuñez, Taylor Adkins, Staci Thomas, Katie Beasley, Tinu Akintobi, Lori Crosby, Susmita Kashikar-Zuck, Carlton Dampier, Gregory D Myer, Trisha Kesar, Charles T Quinn, Soumitri Sil

**Affiliations:** Department of Pediatrics, Emory University School of Medicine, Atlanta, GA, United States; Aflac Cancer and Blood Disorders Center, Children’s Healthcare of Atlanta, Atlanta, GA, United States; Department of Pediatrics, Emory University School of Medicine, Atlanta, GA, United States; Department of Pediatrics, Yale University School of Medicine, New Haven, CT, United States; Department of Rehabilitation Medicine, Emory University School of Medicine, Atlanta, GA, United States; Department of Pediatrics, Emory University School of Medicine, Atlanta, GA, United States; Cincinnati Children’s Hospital Medical Center, University of Cincinnati College of Medicine, Cincinnati, OH, United States; Cincinnati Children’s Hospital Medical Center, University of Cincinnati College of Medicine, Cincinnati, OH, United States; Cincinnati Children’s Hospital Medical Center, University of Cincinnati College of Medicine, Cincinnati, OH, United States; Cincinnati Children’s Hospital Medical Center, University of Cincinnati College of Medicine, Cincinnati, OH, United States; Cincinnati Children’s Hospital Medical Center, University of Cincinnati College of Medicine, Cincinnati, OH, United States; Department of Pediatrics, Emory University School of Medicine, Atlanta, GA, United States; Aflac Cancer and Blood Disorders Center, Children’s Healthcare of Atlanta, Atlanta, GA, United States; Department of Orthopaedics, Emory University School of Medicine, Atlanta, GA, United States; Emory Sports Performance and Research Center, Flowery Branch, GA, United States; Department of Rehabilitation Medicine, Emory University School of Medicine, Atlanta, GA, United States; Cincinnati Children’s Hospital Medical Center, University of Cincinnati College of Medicine, Cincinnati, OH, United States; Department of Pediatrics, Emory University School of Medicine, Atlanta, GA, United States; Aflac Cancer and Blood Disorders Center, Children’s Healthcare of Atlanta, Atlanta, GA, United States

**Keywords:** sickle cell disease, physical activity, adolescents, young adults, qualitative

## Abstract

**Objectives:**

Chronic pain among youth with sickle cell disease (SCD) is associated with significant functional disability. Physical activity is recommended for pediatric chronic pain and is safe, feasible, and beneficial for individuals with SCD, yet uptake is limited. This study describes the adolescent- and caregiver-centered lived experience of physical activity within the context of SCD and chronic pain to inform intervention targets.

**Methods:**

Adolescents aged 12–18 years with any SCD genotype and medium or greater risk of chronic pain (Pediatric Pain Screening Tool) were recruited across two sites for an intervention development study. Semi-structured interviews elicited perspectives related to physical activity and its role in pain management. A deductive-inductive approach was used with the Fear Avoidance Model as an analytic framework.

**Results:**

Adolescents (*n* = 12; 15.1 ± 1.5 years) were Black/African American, even sex distribution, with 92% Medicaid-covered. Caregivers (*n* = 12; 39.4 ± 5.8 years) were Black/African American, and 100% were mothers/stepmothers. Physical activity facilitators included structured social activities and older age. Barriers included triggering or worsening pain and safety concerns expressed by caregivers and adults. Caregivers emphasized that adolescents developing self-awareness helps them modify physical activity to prevent pain. Benefits of physical activity to manage pain included minimizing stiffness and pain exacerbation and sustained pain reduction.

**Conclusions:**

Physical activity to manage chronic SCD pain may require individualization and adaptation to address patient and caregiver concerns. Future intervention targets need to address unique facilitators and barriers, minimize challenges, and promote benefits of physical activity for chronic SCD pain.

Sickle cell disease (SCD) is a family of rare genetic disorders of the hemoglobin affecting approximately 100,000 individuals in the United States, predominantly of African descent. Chronic pain in SCD, defined as pain on most days for 6 or more months, can begin during adolescence, affects ∼20% of youth, and contributes to pain-related functional impairments in daily activities, low rates of school attendance, limited social functioning, and diminished health-related quality of life (Dampier et al., 2017; Poku et al., 2018). These disruptions to daily functioning may be attributed to multiple contributors, including physical inactivity secondary to pain or concerns about causing or exacerbating pain (Olorunyomi et al., 2022). Drawing from literature on other chronic pain conditions (e.g., generalized musculoskeletal pain), engagement in physical activity has promising effects to improve daily functioning, manage pain, and improve social and health-related quality of life among adults (De la Corte-Rodriguez et al., 2024; Geneen et al., 2017). Yet, the evidence of physical activity for pediatric chronic pain conditions remains limited due to a paucity of studies (Leite et al., 2022).

Physical activity encompasses a wide variety of activities (e.g., aerobic exercise, strength training, flexibility) and can occur at a range of intensities and durations in a variety of settings, from unstructured and self-directed activity (e.g., playing outside) to structured and instructor-led activities (e.g., formal physical therapy, exercise classes). For youth with SCD and chronic pain, engaging in physical activity may be further complicated by medical comorbidities (e.g., asthma, acute chest syndrome, avascular necrosis, low bone density), and there is limited guidance on how these comorbidities affect participation or recommendations for physical activity (Connes et al., 2024; Marchese et al., 2021). The need for further research to elucidate the role of physical activity in managing SCD is also evidenced by the lack of empirical support for a clear recommendation regarding physical activity or exercise for youth with SCD in recent American Society of Hematology SCD clinical practice guidelines (Brandow et al., 2020).

A small body of research on physical activity in the context of SCD has yielded preliminary physiological and self-reported data suggesting that it may be safe and feasible for youth with SCD, though with no distinctions regarding chronicity of pain. Youth with SCD report exercising more intensely and for shorter durations than peers without SCD (Omwanghe et al., 2017). High-intensity, maximal cardiopulmonary exercise testing is safe among children and adults with SCD, with <1% experiencing sickle cell-related pain episodes (Smith et al., 2022). Further, low- to moderate-intensity graded aerobic exercise programs can improve exercise tolerance and reduce levels of inflammatory markers in individuals with SCD (Pinto et al., 2021). In children and adults with SCD, stationary bicycle use and exercise training (i.e., muscular, resistance, and flexibility exercises), respectively, are feasible and safe at home (de Almeida et al., 2021; Liem et al., 2017), as are longer-term exercise programs of mild-to-moderate intensity (Connes et al., 2024). Despite these promising findings, there has been limited investigation on the potential benefits of physical activity for chronic SCD pain management, likely due to longstanding unsubstantiated concerns about potential negative impacts and risks of physical activity (e.g., increased vaso-occlusive events) that may perpetuate activity avoidance (Liem, 2018).

The Fear Avoidance Model of Chronic Pain (Leeuw et al., 2007; Vlaeyen & Linton, 2000), developed to explain psychosocial factors in the development and maintenance of chronic pain, offers a framework for organizing previous findings related to the experience of physical activity in the context of pediatric chronic SCD. In this model, following the experience of pain, two possible pathways are theorized. Catastrophic beliefs about pain have the potential to contribute to fear of potential pain, leading to avoidance of possible pain-causing situations or hypervigilance about potential pain. In turn, avoidance and hypervigilance contribute to low mood and diminished functioning, amplifying the negative impact of any future pain experiences and perpetuating this cycle of fear and avoidance. Alternatively, a lack of fear following a pain experience and willingness to confront that situation again is thought to lead to recovery. Fear of movement is associated with general psychological distress among adults with SCD (Pells et al., 2007), which may further reinforce cycles of physical inactivity, pain, and depressive and anxiety symptoms (Karlson et al., 2020). Similarly, fear of pain and disease-related complications are barriers to engaging in physical activity among youth with minoritized identities and chronic diseases such as SCD (Olorunyomi et al., 2022) and may contribute to avoidance of physical activity, greater functional impairment, and increased pain severity, consistent with the Fear Avoidance Model. For children and adolescents with SCD, engagement in physical activity is also embedded within their developmental, familial, and social contexts, which are key considerations for understanding the unique role of physical activity in managing pain and functioning (Palermo et al., 2014). Caregivers of youth with SCD often report high levels of catastrophic thinking about their child’s pain, which can increase the child's risk for functional impairment and depressive symptoms (Goldstein-Leever et al., 2018; Schneider et al., 2022; Sil et al., 2016) and may influence caregiver concerns about their child engaging in physical activities.

As such, a qualitative examination is well suited to advance knowledge regarding fears, barriers, and facilitators related to physical activity in youth with chronic pain and SCD and their caregivers to inform the development of clinical guidelines and multicomponent treatment approaches individualized to maximize benefits and safety (Pinto et al., 2021). This study aimed to describe the adolescent- and caregiver-centered lived experiences of physical activity within the context of SCD and chronic pain to clarify physical and psychological barriers and facilitators of physical activity and thus inform future intervention targets.

## Methods

This study was part of a larger project focused on developing and adapting an integrative, multicomponent, behavioral intervention combining mind–body, cognitive, and behavioral coping skills with neuromuscular exercise training for adolescents with chronic SCD pain. This study presents the first phase of the intervention development process, focusing specifically on patient and caregiver perceptions of physical activity within the context of SCD pain management.

### Participant recruitment

Participants were recruited in June–October 2023 across two comprehensive SCD programs at children’s hospitals in the Southeastern and Midwestern United States. Adolescents were eligible if they had any SCD genotype, were aged 12–18 years, had a medium to high risk of chronic pain on the Pediatric Pain Screening Tool, and were receiving stable standard care (including disease-modifying medications or treatments, if applicable) for the past 3 months. Interpretive services were available in both recruitment settings for potential participants who were most comfortable in a language other than English, although no participants required this service. Exclusion criteria included significant documented cognitive or developmental disabilities that would interfere with participation or comorbid medical conditions in which pain is common (e.g., rheumatological or gastrointestinal conditions). Purposive sampling was used to identify potential participants at each site and obtain a sample that was representative across adolescent ages and medium versus high risk of chronic pain.

### Procedures

This study was approved by the Western Clinical Group Institutional Review Board prior to study initiation. Adolescents were pre-screened for eligibility and approached by a consenting study team member during clinic visits or by phone. Consenting study team members were primarily those with prior experience with research with this population and ongoing, regular contact with potential participants. Adolescents and caregivers were provided an in-depth explanation of the study with visual infographics, including the researchers’ interest in and goals for the research, and those interested in participating provided written assent and consent, respectively, during the clinic visit or by phone. Young adults 18 years of age were eligible to enroll without a caregiver and provided written consent. Caregivers (or young adults enrolling independently) completed a brief battery of measures on sociodemographic characteristics either electronically via REDCap or by paper-and-pencil questionnaire, based on their preference.

A semi-structured interview guide was developed in collaboration with a community advisory board consisting of community health workers and persons with lived experience of SCD. The interview guide included open-ended questions about adolescents’ perceived barriers and facilitators of physical activity within the context of SCD and perspectives of physical activity for SCD pain management, such as “What concerns, if any, do you have about doing physical activity?, How does physical activity help with pain, if at all? Tell me about any time when your teen has wanted to play sports or other activities. How did it go? What are your concerns about this, if any?” Interviews were conducted by C.S., a cisgender female PhD-level researcher with expertise in qualitative methodologies with no prior contact with the participants. The adolescent–caregiver dyad was interviewed jointly or separately, based on family preference, and lasted 30–45 min during one or two interview sessions, conducted over secure videoconferencing software to support rapport building and connection between the interviewer and participants while minimizing travel and financial burden to the participants. Second interviews were conducted if time constraints limited participants’ completion of initial interviews or to follow up on additional probes that were informed by ongoing evaluation of interview transcripts. During interviews, the interviewer briefly reviewed the purpose of the research and the researchers’ interest and goals related to the topic area. Interviewer and study team members managed personal influence on the data analysis process by discussing thoughts, impressions, and potential biases before and after conducting interviews. The interviewer made brief field notes during and after interviews to facilitate later analysis and inform ongoing evaluation of new ideas or insights prompting further exploration. Interviews were audio-recorded, transcribed verbatim by a professional transcription service, and de-identified at the time of transcription. Transcripts were reviewed for accuracy against the audio recording by a study team member and were not reviewed by participants directly to minimize participant burden. All participants received compensation for their time participating in the study.

### Measures

#### Pediatric Pain Screening Tool

The Pediatric Pain Screening Tool (PPST) (Simons et al., 2015) is a 9-item self-report measure of functional disability and psychosocial distress associated with pain in youth, used to indicate risk of chronic pain (low, medium, or high), and has demonstrated good psychometric properties among youth with chronic pain, headaches, and SCD. The PPST ranges from 0 to 9, and scores >3 (suggesting medium or high risk; Sil et al., 2018) were used to assess study eligibility.

After evaluation of eligibility, the following measures were completed by caregivers to minimize participant burden.

#### Demographics

A demographic survey collected information regarding adolescent sex assigned at birth, ethnicity, race, caregiver highest education, caregiver employment, caregiver relationship status, and annual household income. Zip codes were used to derive rural–urban commuting area (RUCA) codes (US Postal Service et al., 2021), which classify US census tracts using measures of population density, urbanization, and daily commuting. Codes are scored 1–10, where 1 indicates an urbanized metropolitan area and 10 indicates a rural area.

#### PhenX toolkit

Standard measures from the PhenX Toolkit SCD protocol were used to gather information on insurance coverage (PhenX Toolkit, 2023b) and frequency of SCD pain episodes per year (PhenX Toolkit, 2023a).

#### Physical activities

Caregivers rated their adolescents’ level of physical activity on a 0–10 Likert scale (0 = *not at all active*; 10 = *extremely active*) and reported common types of physical activities enjoyed by their adolescents. This item was developed informally in the context of this study based on other common 10-point numeric rating scales (e.g., numeric pain rating scale) to facilitate relevant prompting and probes during dyadic and individual interviews. Caregivers also provided a history of the adolescents engaging in physical therapy for SCD management.

### Data analysis

Transcripts were analyzed using a hybrid deductive-inductive framework approach (Gale et al., 2013) incorporating elements of coding-reliability thematic analysis within a primarily positivist (i.e., “small q”) qualitative framework (Braun & Clarke, 2023). Deductive analysis was shaped by the elements of the Fear Avoidance Model of Chronic Pain, and centered on physical and psychological barriers to and facilitators of physical activity within the context of SCD and chronic pain. Building on this initial framework, we integrated an inductive approach through ongoing open coding to facilitate the identification of specific barriers and facilitators relevant to physical activity in the context of SCD and chronic pain. Thus, the resulting shared concepts are best termed as topic summary themes (Braun & Clarke, 2023, p. 3). After familiarization with the transcripts, three coders with experience in qualitative methods (C.S., S.S., J.T.M.), including a content expert in pain in pediatric SCD (S.S.) identified segments relevant to the research questions and grouped these and similar responses under codes (labels that broadly summarized the interpreted meaning, e.g., “participated in organized sports”), using NVivo software (QSR International, 2020). Codes and their associated data were re-reviewed by the primary analyst (C.S.) to organize them into overarching shared concepts (e.g., “facilitators of physical activity”), which were refined through additional discussion and re-review of the data with an additional content expert (N.B.) to develop final themes. Approximately 25% of transcribed interviews were independently coded by two study team members (C.S., J.T.M.) to ensure inter-rater reliability of the final codebook, yielding ≥90% agreement. Participants were not asked to re-review the data to minimize participant burden. The data underlying this article are subject to an embargo until date of publication. After publication, the data will be available in Vivli data repository.

## Results

### Participant characteristics

A total of 36 participants (*n* = 19 adolescents, *n* = 17 caregivers) provided consent, and 24 participants (*n* = 12 adolescents, *n* = 12 caregivers) completed in-depth interviews across both sites. Six teen–caregiver dyads (*n* = 12) completed interviews over two sessions to ensure saturation of additional themes that emerged from ongoing data analysis. Non-participation was primarily attributed to participants unable to be contacted for scheduling. There was no significant differential attrition for participation based on age, sex assigned at birth, race, ethnicity, pain screening risk score, RUCA code, or caregiver-reported level of physical activity. Adolescents had a mean age of 15.1 years [standard deviation (*SD*) = 1.5], and caregivers were 100% mothers/stepmothers with a mean age of 39.4 years (*SD* = 6.3). About 80% of adolescents experienced at least four vaso-occlusive pain episodes in the past 6 months that limited their daily activity, and 86.7% missed at least 1 week of daily activities due to pain. Table 1 provides the distribution of demographic characteristics of adolescents and caregivers.

**Table 1.** Sample characteristics [n = 24 (12 children, 12 caregivers)].

	% (or *mean*)	N (or *SD*)
Child		
Age, years	*15.17*	*1.53*
Sex assigned at birth		
Female	50%	6
Male	50%	6
Race		
African American	100%	12
Ethnicity		
Non-Hispanic/Latino	100%	12
Hemoglobin (Hb) type		
HbSS or HbSB^0^-thalassemia	91.67%	11
HbSC or HbSB^+^-thalassemia	8.33%	1
Presence of avascular necrosis	25%	3
Emergency department visits for pain		
Past 6 months	*2.69*	*3.05*
Past 12 months	*4.19*	*4.65*
Caregiver		
Age, years	*39.42*	*6.30*
Reporter		
Mother/stepmother	100%	12
Race		
African American	100%	12
Ethnicity		
Non-Hispanic/Latino	91.67%	11
Unknown/not reported	8.33%	1
Marital Status		
Married/partnered	50%	6
Never married	25%	3
Divorced/separated	25%	3
Widowed	0%	0
Highest grade completed		
High school or secondary school	50%	6
Associate’s or technical degree	25%	3
College degree	25%	3
Annual family income		
≤$10,000	8.33%	1
$10,000–$24,999	16.67%	2
$25,000–$34,999	33.33%	4
$35,000–$49,999	8.33%	1
$50,000–$74,999	16.67%	2
$75,000–$99,999	8.33%	1
Prefer not to answer	8.34%	1
Insurance coverage^a^		
Medicaid/managed care organization		15
Medicare		2
Private		5

*Note.* All participants reported a gender identity that was concordant with their sex assigned at birth. Medicaid and Medicare refer broadly to a set of US public insurance programs supported by both federal and state funding, sometimes managed by a third-party organization, and with specific medical/disability, age-related, and/or financial eligibility criteria. Private insurance refers to insurance that is funded by a caregiver’s employer or by the caregiver (e.g., purchased through an insurance coverage marketplace). Patient financial responsibility and procedure/treatment coverage varies across public and private and within private insurance carriers. Percentage is indicated with the % sign, whereas mean values are in italics (as in the header), and number is differentiated from standard deviation similarly (standard deviations are indicated in italics).

a Insurance coverage categories are not mutually exclusive—some participants reported more than one type of coverage.

Using the general structure of the Fear Avoidance Model as a framework, the analysis broadly focused on facilitators and barriers of engaging in physical activity as reflections of the two major pathways (pain catastrophizing and fear leading to avoidance and disability; lack of fear leading to confrontation and recovery). No further structure was applied for specific facilitators and barriers to allow for variability and concepts unique to this population. Adolescents and caregivers described two overarching themes: (1) patterns of past and current physical activity, including barriers and facilitators of engaging in physical activity, and (2) benefits and challenges of physical activity and exercise to manage SCD pain.

### Factors contributing to past and current physical activity

Adolescents’ past and current engagement in a range of activities were associated with various barriers and facilitators (see Table 2 for representative quotes).

**Table 2.** Representative quotes of factors contributing to past and current physical activity.

Theme: Development of self-management skills
“I guess it's a proactive caution. [Laughter] With everything, because <Participant> she older now. When she was younger, I used to restrict her from a lot of things because I didn't feel like she was able to communicate effectively. Now she's 13, she's able to—she's more independent now. She can tell. Now, actually, she said she can feel when a pain crisis is coming so it just gives me a little bit more trust that she's being more aware of her own condition versus just well, let me just listen to what mommy say.” (Caregiver of 13-year-old female)
“Yes, like just her being a typical teenager. I’m a little more laid-back now that she’s older and understands her conditions, but while she was growing up and not really understanding what it was that she was facing, and wanting to go swimming and just go do other things with the kids, that I have to remind her, “Hey, you can’t stay in the pool too long,” or tell the other parents, “Hey, can you keep an eye out for this,” or just certain instructions that you have to give.” (Caregiver of 16-year-old female)

Theme: Exacerbating SCD symptoms
“Yes [affirms worrying]. If I try to run or play sports that I'll injure myself, or I overwork myself to the point where I'll probably end up in the pain crisis the next day.” (13-year-old female) “The only thing I think prevents me from doing exercises is pain, especially when it rains.” (15-year-old male)
“I will say that sickle cell has limited him. He used to play sports. Because of sickle cell, it stripped it away from him.” (Caregiver of 17-year-old male)
“No [denies worrying]. I just know that sometimes I’m gonna have pain afterwards.” (15-year-old female)
“I feel fine, but I learned how to pace myself while doing it, so I won't overdo it.” (17-year-old female)

Theme: Caregiver worry
“Like, all of it terrified me ‘cause I didn’t know what to expect. Like, over these years, I’ve learned, all right, his pain places are like this, in this area of his body. If he does this too long or he’s in the pool or whatever, like, just small stuff that people probably don’t pay attention to…” (Caregiver of 13-year-old male)
“I don't worry as much, but it's still in the back of my mind too. She started playing tennis, so then I was, well, you gotta be careful on your movements, because if you move too fast trying to chase the ball, there could be a possibility that you could pop your hip out of socket. Things like that that's always on my mind.” (Caregiver of 13-year-old female)
“My main thing I would just tell other parents is to trust your children. They will understand and learn their limits, but just don’t stop them from being a kid. If they want to cheer or play basketball, or if there’s anything that they want to do, allow them to do it and experience it, and learn what they can do, I guess, with their condition. I don’t want them to stop their children from being kids, ’cause that can kinda suck.” (Caregiver of 16-year-old female)
“I would say don’t put your fears on your child. You don’t know what they are capable of doing until they try.” (Caregiver of 13-year-old male)

Theme: Restrictions from other adults
“In middle school, they [gym teacher] discouraged me from doing like the PACER test [Progressive Aerobic Cardiovascular Endurance Run].” (18-year-old male)
“Yeah [affirms discouragement from other adults], with football. [Who told you, you couldn’t play?] The doctor.” (18-year-old male)
“Yes [affirms discouragement from other adults]. Because of my health. Probably my teacher, gym teachers. Some of my teachers, they know I have sickle cell so when they try to plan stuff, they all try to plan it on a good day so it's not too hot or not too windy.” (13-year-old female)

#### Facilitators of physical activity

Participants expressed two primary facilitators that support physical activity: social interaction and the development of self-management skills. Within the context of the Fear Avoidance Model, facilitators of physical activity might exemplify factors that minimize fear and support confrontation and recovery from a pain experience.

##### Social interaction

On average, caregivers reported that their teens were moderately physically active [*M* = 5.2, *SD* = 2.5; scale: 0 (not active at all)–10 (extremely active)]. Participants’ physical activities included regular participation in organized sports (e.g., basketball, football, soccer, volleyball; 19%), structured physical activities (e.g., gymnastics, dance, gym class; 38%), unstructured physical activities (e.g., bicycling, playing outside, walking; 63%), and limited to no physical activity due to lack of interest or perceived inability to engage in physical activities due to disease-related limitations (6%). Most adolescents (63%) had participated in physical therapy in the past during hospitalizations and/or in outpatient settings.

Adolescents who engaged in some form of physical activity often described the benefits of social interaction with friends or peers during activities. Social interaction is likely a key motivator for teens to engage in physical activity to facilitate connections with peers, friends, and family members.

##### Development of self-management skills

Caregivers expressed that their child typically participated in more physical activity as they aged into adolescence, which was primarily attributed to the development of disease self-management skills. Although caregivers generally preferred not to completely restrict their child’s physical activity, they described limiting younger children’s activities due to their lack of awareness of the risks of overexertion, limited knowledge of how to manage their disease, and budding communication skills to articulate their self-management needs. Consequently, caregivers outlined frameworks to inform their younger children on when to stop or avoid an activity, incorporating factors such as temperature, weather, or intensity level, that were more directive for their child’s involvement in physical activity. With the transition to adolescence, caregivers felt more comfortable allowing their teen to self-manage their physical activity. This was consistent with adolescents’ own perception of their improved self-management, specifically learning their limits and pacing their activities.

#### Barriers to physical activity

The most common barriers to physical activity were exacerbating SCD symptoms, caregiver worry, and restrictions from other adult authority figures. Within the Fear Avoidance Model, barriers to physical activity might include pain-related catastrophizing and fear related to prior pain experiences.

##### Exacerbating SCD symptoms

Most commonly, participants reported that SCD-related factors prevented or restricted their physical activity. Most adolescent participants had experienced SCD-related complications, such as acute chest syndrome or SCD-related pain that prevented them from fully engaging in physical activities or led to them discontinuing their organized sports. Some adolescents expressed fear that participation in physical activities or physical overexertion might trigger or exacerbate pain and fatigue, whereas other adolescents acknowledged and may have accepted pain as a possibility after physical activity. Adolescents noted practice, pacing, and modification as ways to manage the risk of developing pain following activity.

##### Caregiver worry

Caregivers expressed similar perspectives regarding potential pain as a barrier to engaging in physical activity, centering on the importance of recognizing and appropriately modifying physical activity when needed to prevent pain. Although caregivers expressed worries about their child overexerting themselves and potentially eliciting increased pain, caregivers generally believed they should teach their adolescent how to manage their SCD with a balanced approach.

##### Restrictions from other adult authority figures

Beyond limitations that may be placed by their caregivers, adolescents expressed that other adult authority figures in their lives were concerned about their physical activity to the extent that it limited their engagement. Examples included gym teachers discouraging participation in gym class, asking them to sit aside, requiring a doctor’s approval to play sports, as well as physicians discouraging participation in sports. For other adolescents, teachers and coaches worked with the adolescent to allow them to safely participate in physical activities.

### Perceptions and experience with physical activity for pain management.

Adolescents’ perspectives about the role of physical activity to help manage pain considered both benefits and challenges, ranging from viewing physical activity as an essential element of chronic pain management to expectations that it will worsen pain (see Table 3 for representative quotes). Applied to the Fear Avoidance Model, conceptualizing physical activity as a way to manage or treat pain would likely facilitate continued engagement with physical activity, whereas conceptualizing physical activity as a risk for exacerbation or worsening of pain would have the potential to lead to avoidance and further disability.

**Table 3.** Representative quotes of perceptions and experiences with physical activity for pain management.

Theme: Fear of pain and overexertion
“When I have a pain crisis, I don’t do nothing. 'Cause it’s impossible for me to move.” (17-year-old male)
“I’d rather stay still than moving the body, because at times it does make me think that stay still is better.” (16-year-old female)
“But if his pain level is at a seven, no, lay there because that’s how we end up in the ER you keep moving around. But once it dies down to like a two, you know, he likes to walk around and check on everybody. But he still has a heat pad close up though.” (Caregiver of 16-year-old male)

Theme: Activity mitigates pain
“I'll walk around, or I'll pace or I move my joints. Things like that, or I'll stretch just to make my blood flow because my blood, it gets sticking.” (13-year-old female)
“I believe they can feel the onset of it [pain episode], so before it gets too severe or to a full-blown crisis—if, let’s say, if her arm is hurting, I’ll try to tell her, ‘Don’t let it stiffen up on you. Try to move it around or massage’” (Caregiver of 16-year-old female)
“A lot of their behavior is, if I just sit still, I can get over this pain, and then I can get back to my normal. Then when you try to sit still all day, or two, three days, and now it's time for you to do your exercises, you're gonna be in pain because you wasn't moving or practicing your exercises before.” (Caregiver of 15-year-old male)
“That is our theory in our house. If you act sick, you feel sick. Yeah. The first couple of days we'll let him just lay around. By the third day, we try to get him to move and stretch…I truly believe that movement is definitely one of the things that stops the pain.” (Caregiver of 17-year-old male)
“Sometimes, my son would keep walking. He made it up to 16 minutes walking. He was in a wheelchair from 3 to 14, so this is his first year out of the wheelchair. He found, oh, if I keep walking, my back will feel better.” (Caregiver of 15-year-old male)

#### Fear of pain and overexertion

Some adolescents expressed concern about how physical movement might worsen their pain and considered pain a sign that they should remain sedentary or still. Caregivers also described a threshold for pain where they discouraged their child from any movement. As the pain episode increased in intensity, caregivers and teens both expressed fear that movement might worsen pain.

#### Activity mitigates pain

Adolescents described using physical activity such as stretching and walking to lessen their pain when they felt the onset of a pain episode. Similarly, caregivers reported that when their child experienced the onset of a pain episode, they generally encouraged mild activity (e.g., stretching, tensing/relaxing, gentle movements) to prevent or minimize stiffness and recognized the benefits of movement and activity to minimize further pain exacerbation. Caregivers also recognized that gradual or continued engagement in physical activity improved their teen’s chronic SCD pain management over time.

## Discussion

There is an urgent need to identify novel and effective treatment approaches for youth with chronic SCD pain. In other chronic pain conditions, moderate physical activity has been shown to support chronic pain management by reducing pain (Guddal et al., 2017), suggesting potential applicability for youth with SCD. However, translation of these benefits via tailored interventions and exercise prescription for youths with SCD requires understanding of specific barriers and facilitators to physical activity. This study fills a scientific gap by representing the lived experiences of adolescents with chronic SCD pain and their caregivers to illustrate diverse influences on engaging in physical activity and its role in managing chronic SCD pain.

Despite empirical evidence suggesting safety, tolerability, and potential benefit of physical activity (Connes et al., 2024), teens, caregivers, and their reports of other adult authority figures suggested persistent and widespread concerns about the safety of movement for individuals with SCD. Consistent with the Fear Avoidance Model of Chronic Pain (Leeuw et al., 2007; Vlaeyen & Linton, 2000), adolescents and caregivers both expressed worries about physical activity triggering or exacerbating pain and implemented physical activity restrictions or avoidance to prevent pain. This perspective may reflect strategies that are adaptive in the setting of acute, episodic pain. However, for youth with chronic pain, this pattern of activity avoidance may contribute to physical deconditioning and heightened sensitivity to pain. Previous work has linked higher physical activity with lower pain among youth with SCD (Karlson et al., 2020) and other adolescent chronic pain conditions (Rabbitts et al., 2014), emphasizing that the fear-based pain avoidance observed in our study also may act as a barrier to experiencing benefits of physical activity and may increase risk for functional disability. This pattern is consistent with the catastrophizing and fear-based pathways toward disability outlined in the Fear Avoidance Model, yet also reflects unique considerations for this population, such as the prior experience of SCD-related complications, as exemplified by a 13-year-old female teen, “Yes [affirms worrying]. If I try to run or play sports that I'll injure myself, or I overwork myself to the point where I'll probably end up in the pain crisis the next day.”

Caregiver concerns emerged as a significant regulator of past and current physical activity in youth with SCD, consistent with previously observed links between elevated parental concerns about pain and increased functional disability in youth with SCD, even when the youth themselves exhibit relatively low worry or concern (Sil et al., 2016). From a developmental perspective, caregiver perspectives and concerns are key considerations affecting physical activity patterns and decisions in youth with SCD (Palermo et al., 2014). In this study, caregivers described developmental increases in their trust in older teens’ self-management of pain, contrasting prior work focused on broad self-management for adolescents with SCD that emphasized parents’ reluctance and perceived risk associated with allowing adolescents to self-manage (Kayle et al., 2016).

Social interaction and age-related independence emerged as facilitators of physical activity, consistent with previously reported significance of social interaction for youth with SCD as a motivator to participate in physical activity (Olorunyomi et al., 2022). Adolescents were more likely to engage in physical activities when accompanied by peers rather than alone, with structured activities such as cheerleading providing both engagement opportunities and valuable social connections. Within the context of the Fear Avoidance Model, it may be that social facilitation provides opportunities to confront activities and supports recovery. Notably, adolescents with SCD describe a more active role in their disease management as they age, which is accompanied by mild worry, conflict with parent concerns, and reluctance to share with peers (Kayle et al., 2016), which may interfere with social engagement.

Our findings offer improved recognition of barriers, facilitators, and the developmental context of self-management in youth with chronic SCD pain. The sample is representative of variability among youth with chronic SCD pain in age, sex assigned at birth, and caregiver and family socio-demographics. Yet our findings are tempered by some study limitations. All of the caregivers who participated in this study were mothers. Thus, direct perspectives of other caregivers, healthcare providers, or other adult authority figures were not included. Future work could explore providers’ beliefs and recommendations regarding physical activity for chronic SCD pain management and intentionally recruit other caregivers to extend beyond patient and maternal perspectives. Additionally, the sample may overrepresent youth with chronic SCD pain who are already interested in physical activity or physical activity-based interventions to manage chronic SCD pain. Future research may consider less-conservative eligibility criteria across the continuum of acute-to-chronic pain to elicit broader perspectives on physical activity as it relates to the transition from acute-to-chronic pain in SCD. Future studies also may consider alternative, nonverbal methods of data collection (e.g., picture sorting/choice) to expand inclusion of those with cognitive or developmental disabilities who would be unable to participate in an interview.

There are multiple interacting familial, social, and physical environmental factors impacting decisions about physical activity for youth with chronic SCD pain, yielding a variety of perspectives that exemplify possible drivers of the divergent pathways in the Fear Avoidance Model. Firsthand accounts from caregivers and youth with chronic SCD pain underscore the importance of population-specific and individualized exercise prescriptions refined through collaborative decision-making. From a developmental perspective, gradual improvement of self-management skills may mitigate caregiver concerns and promote increased confidence for youth with chronic SCD pain and their caregivers regarding their pain management. Future work in exercise and physical activity for youth with chronic SCD pain also should carefully consider peer networks, which may facilitate physical activity engagement. To produce feasible, acceptable, and effective physical activity interventions for youth with chronic SCD pain, intervention development should integrate quantitative knowledge about the safety of gradually approached, low- to moderate-intensity exercise, breaks, and frequent hydration (Connes et al., 2024) with qualitative knowledge regarding fear of pain and complications, the importance of individualization and collaboration, and the developmental context of adolescence. Future intervention development could include a group component to incorporate peer networks, involve parents periodically to support self-management and consideration of familial context, cognitive-behavioral interventions to manage fear of pain and avoidance of activity, and modifiable, moderate-intensity exercises with integrated breaks and hydration to support youth to gradually increase activity in the unique context of SCD and chronic pain.

In the interim, within clinical settings, clinicians may consider intentionally verbalizing their recognition of the fear and concern for complications that may arise related to physical activity for youth with chronic pain and SCD and their families. To help patients and families balance their consideration of medical/physiological risk of complications and the importance of physical activity for functioning, clinicians might inquire about prior experiences in physical activity and promote paced engagement in highly valued activities. Continued education on the emerging evidence of the safety of exercise testing in individuals with SCD along with the risks associated with underactivity and avoidance is warranted to help inform the individualized risk/benefit profile of physical activity. Integrating patient and family knowledge of the patient’s personal symptom presentation and experience of SCD complications is also likely to be important to developing a realistic and safe plan for physical activity with appropriate safeguards.
